# P-874. Examining Outpatient Antibiotic Prescribing Trends and Guideline Concordance Across Urban and Rural Healthcare Facilities

**DOI:** 10.1093/ofid/ofaf695.1082

**Published:** 2026-01-11

**Authors:** Kaylee Caniff, Michael Klepser, Kushal Dahal, Madelyn Koski, Jenna Wilkerson, Minji Sohn

**Affiliations:** Ferris State University, Grand Rapids, Michigan; Ferris State University, Grand Rapids, Michigan; Ferris State University, Grand Rapids, Michigan; Ferris State University, Grand Rapids, Michigan; Ferris State University, Grand Rapids, Michigan; Ferris State University, Grand Rapids, Michigan

## Abstract

**Background:**

Urban and rural outpatient care settings may exhibit differences in antibiotic prescribing patterns due to factors such as accessibility, provider practices, and patient demographics. Using a multi-system registry, we examined antibiotic prescribing patterns in Michigan's urban and rural outpatient settings to inform antimicrobial stewardship targets.

**Table 1.**
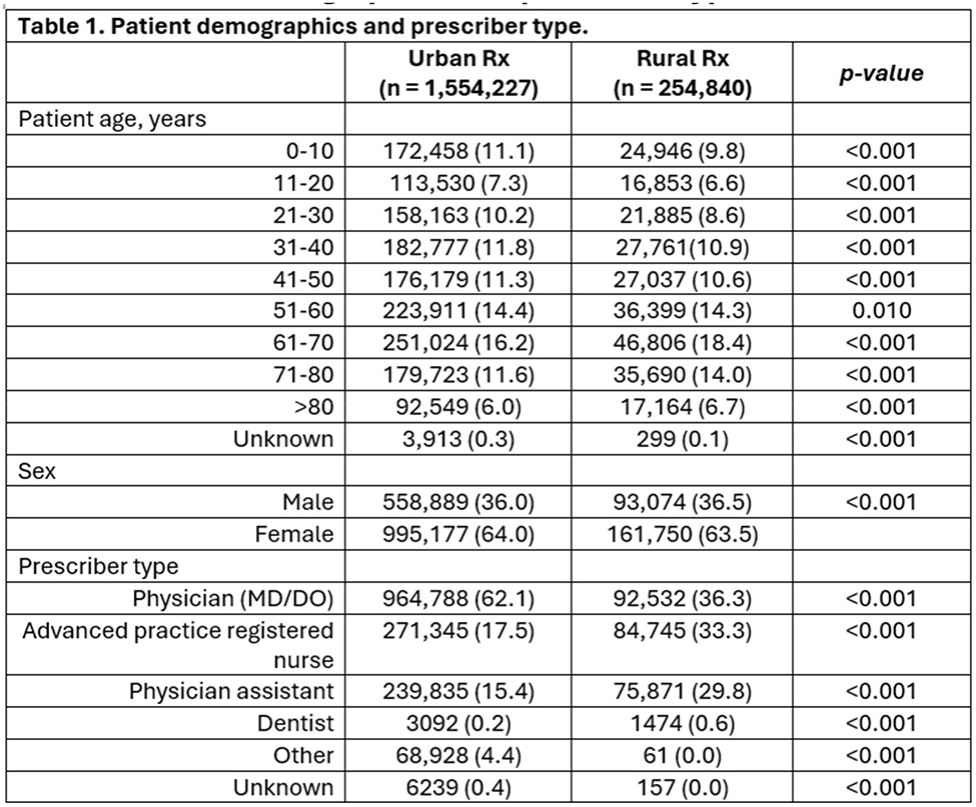
Patient demographics and prescriber type.

**Figure 1.**
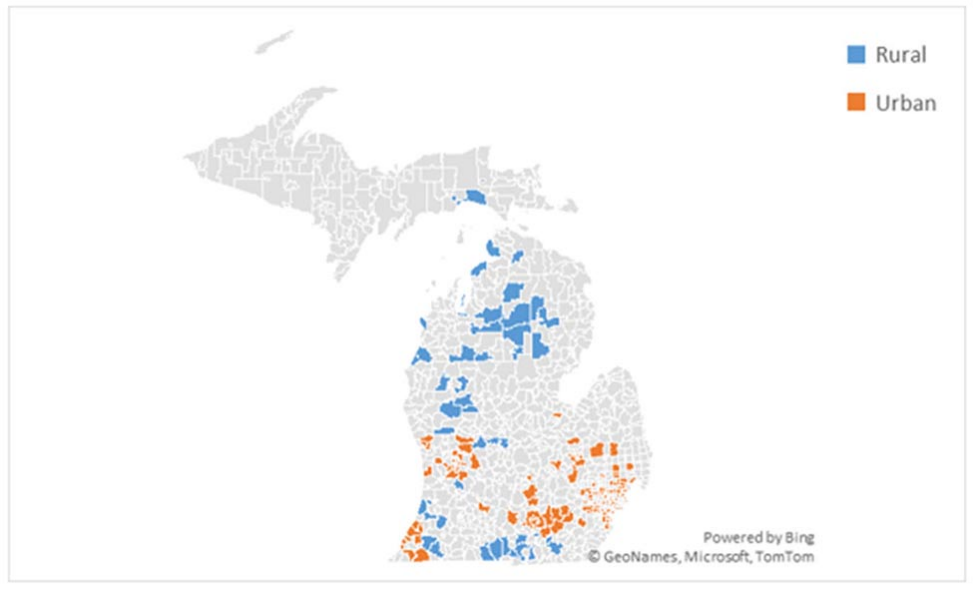
Geographic distribution of included facilities by ZIP code and rurality.

**Methods:**

The Collaboration to Harmonize Antimicrobial Registry Measure (CHARM) database was utilized to analyze antibiotic prescriptions and associated outpatient encounters from participating Michigan facilities from 1/2020-12/2024. Healthcare facilities were categorized as rural or urban by ZIP code according to the U.S. Census Bureau definition. Data collected included patient demographics, prescriber type, ICD-10 code, and antibiotic regimen. Appropriateness of antibiotic and dosing regimens were assessed for concordance with diagnosis-specific guidelines as appropriate.

**Figure 2.**
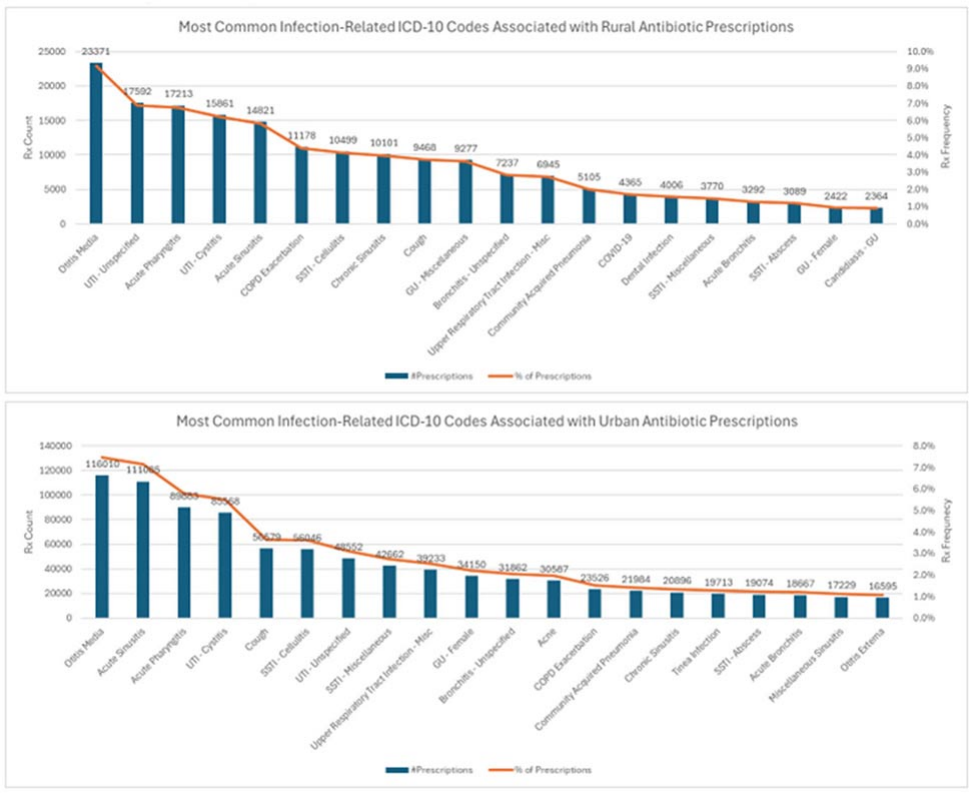
Most common infection-related ICD-10 codes associated with urban and rural antibiotic prescriptions.

**Results:**

A total of 1,554,227 antibiotic prescriptions were linked to 942,991 patient encounters at urban sites (1,057 facilities), and 254,840 prescriptions to 207,208 encounters at rural sites (144 facilities). Physicians prescribed antibiotics most often at urban sites; whereas rural sites had antibiotics commonly prescribed by mid-level providers. Overall, antibiotic prescriptions were more frequently prescribed within outpatient encounters at urban sites (175.4 per 1,000 patient encounters) compared to rural sites (145.4 per 1,000 patient encounters, p < 0.001). Availability of ICD-10 codes and regimen details allowed for the assessment of agent selection for n = 611,068 prescriptions and dosing for n = 300,801 prescriptions. Among prescriptions from urban sites, 68.0% and 63.1% were guideline-concordant for agent and dosing, respectively, compared to 65.7% (p < 0.001) and 62.4% (p = 0.001) of prescriptions at rural sites.

**Conclusion:**

Outpatient antibiotics were prescribed more frequently, but with higher rates of concordance with guidelines, at urban care centers. Future analyses will examine drivers of this outcome as well as independent associations of patient- and prescriber factors with guideline discordant prescribing in urban and rural communities.

**Disclosures:**

All Authors: No reported disclosures

